# Assistenz bei der Selbsttötung in Deutschland – erste Auswertung von Daten einer Berichtsplattform und mögliche Implikationen für die Entwicklung einer Leitlinie

**DOI:** 10.1007/s00103-026-04196-9

**Published:** 2026-02-09

**Authors:** Jan Schildmann, Thomas Pollmächer, Alfred Simon, Sabine Sommerlatte, Georg Marckmann

**Affiliations:** 1 Institut für Geschichte und Ethik der Medizin, Profilzentrum Gesundheitswissenschaften, Medizinische Fakultät, Martin-Luther-Universität Halle-Wittenberg, Magdeburger Straße 8, 06112 Halle (Saale), Deutschland; 2 Donau-Altmühl-Kliniken, Ingolstadt, Deutschland; 3 Klinik für Psychiatrie und Psychiatrie, LMU Klinikum, München, Deutschland; 4 Akademie für Ethik in der Medizin und Institut für Ethik und Geschichte der Medizin, Universitätsmedizin Göttingen, Göttingen, Deutschland; 5 Institut für Ethik, Geschichte und Theorie der Medizin, Ludwig-Maximilians-Universität München, München, Deutschland

**Keywords:** Suizidassistenz, Ethik, Leitlinien, Freiverantwortlichkeit, Beratung, Assisted suicide, Ethics, Guidelines, Counselling

## Abstract

**Einleitung:**

Nach einem Urteil des Bundesverfassungsgerichts vom 26.02.2020 ist die Assistenz bei der Selbsttötung rechtmäßig, wenn sie auf einer freiverantwortlichen Entscheidung der betroffenen Person beruht. Bislang gibt es wenige empirische Untersuchungen zur aktuellen Praxis des Umgangs mit Anfragen nach assistierter Selbsttötung in Deutschland.

**Methoden:**

Seit dem 01.11.2024 können Fallberichte zu Anfragen nach Assistenz bei der Selbsttötung sowie zu durchgeführten assistierten Selbsttötungen auf einer Berichtsplattform (Bericht- und Lernsystem) anonym eingegeben werden. Die quantitativen Daten wurden deskriptiv analysiert. Die Freitexte wurden inhaltsanalytisch ausgewertet.

**Ergebnisse:**

Von den 206 Eintragungen im Bericht- und Lernsystem konnten nach Anwendung der Ausschlusskriterien 133 Fallberichte ausgewertet werden. Die häufigsten Gründe für Anfragen und durchgeführte assistierte Selbsttötungen waren Sorgen vor dem Verlust der Selbstbestimmung (*n* = 33) und der Selbstständigkeit (*n* = 20). In 22 Fallberichten wurden Angaben zur assistierten Selbsttötung und in weiteren 18 Fallberichten zu einer geplanten assistierten Selbsttötung gemacht. In die Prüfung der Freiverantwortlichkeit waren am häufigsten Ärzt*innen und Mitarbeitende von Sterbehilfeorganisationen einbezogen. In 7 Fallberichten wurde angegeben, dass 2 unabhängige Personen die Freiverantwortlichkeit geprüft hatten. In Bezug auf bestehende Bedarfe wurden klare rechtliche Regelungen (*n* = 33) und fachliche Leitlinien (*n* = 20) am häufigsten genannt.

**Diskussion:**

Ausgehend von den erhobenen Daten werden inhaltliche und prozedurale Aspekte möglicher Leitlinienempfehlungen für die Prüfung der Freiverantwortlichkeit und Beratung bei Anfragen nach assistierter Selbsttötung erörtert.

## Abstract (en)

**Introduction:**

According to the ruling of the Federal Constitutional Court of 26 February 2020, assisted suicide is legal if it is based on a freely made decision (“Freiverantwortlichkeit”) by the person concerned. To date, there have been few empirical studies on the current practice of dealing with requests for assisted suicide in Germany.

**Methods:**

Since 1 November 2024, case reports on (requests for) assisted suicide and on the practice of assisted suicide can be entered anonymously on an electronic platform. Descriptive analysis of quantitative data and content analysis of qualitative data.

**Results:**

Of the 206 entries, 133 case reports on (requests for) assisted suicide could be included for further analysis. Concerns about loss of self-determination (*n* = 33) and independence (*n* = 20) were the most frequently cited reasons for requesting assisted suicide. In 22 case reports, information was provided on assisted suicide, and in a further 18 case reports, on planned assisted suicide. Doctors and employees of euthanasia organizations were most frequently involved in assessing decisional capacity and further criteria for legal assisted suicide. In seven case reports, it was stated that two independent persons were involved in the assessment. With regard to existing needs, clear legal regulations (*n* = 33) and professional guidelines (*n* = 20) were most frequently mentioned.

**Discussion:**

Based on the data collected, substantive and procedural aspects of possible guideline recommendations for assessing “Freiverantwortlichkeit” and providing counseling in cases of requests for assisted suicide are discussed.

## Einleitung

Nach einem Urteil des Bundesverfassungsgerichts vom 26.02.2020 ist die Assistenz bei der Selbsttötung rechtmäßig, wenn die Entscheidung über die Selbsttötung freiverantwortlich gefällt wird [1]. Die im Urteil sprachlich nicht durchweg einheitlich gefassten Anforderungen an freiverantwortliche Entscheidungen umfassen 1. die Einsichts- und Urteilsfähigkeit des Sterbewilligen [1, Rn. 249], 2. die Kenntnis aller entscheidungserheblichen Gesichtspunkte [1, Rn. 242], 3. keine unzulässige Einflussnahme oder Druck [1, Rn. 243] sowie 4. die Dauerhaftigkeit und innere Festigkeit des geäußerten Wunsches nach assistierter Selbsttötung [1, Rn. 244]. Nach dem gescheiterten Versuch einer Gesetzgebung 2023 im Deutschen Bundestag bilden die Ausführungen im Urteil des Bundesverfassungsgerichts den zentralen normativen Referenzpunkt für die rechtmäßige Praxis der assistierten Selbsttötung. 2025 hat der Bundesgerichtshof die Revision eines Urteils verworfen, entsprechend dem ein Arzt zu einer 3‑jährigen Haftstrafe wegen Totschlags in mittelbarer Täterschaft verurteilt wurde [2]. Ausgangspunkt für die Anklage war die assistierte Selbsttötung eines psychisch erkrankten Patienten und die Einschätzung des Gerichts, dass die Freiverantwortlichkeit in diesem Fall nicht angemessen geprüft wurde und nicht gegeben war [3].

Die Gesamtzahl der Todesfälle durch assistierte Selbsttötung wird derzeit in Deutschland nicht systematisch erfasst. Verfügbare Daten deuten jedoch darauf hin, dass die Zahlen seit 2020 deutlich angestiegen sind. Im Jahr 2021 berichtete die Deutsche Gesellschaft für Humanes Sterben (DGHS) über 120 Fälle, während für 2024 über 623 Fälle berichtet wird [4]. Gleich et al. [5] untersuchten für den Münchner Raum 77 Fälle assistierter Selbsttötung in den Jahren 2020 bis 2023, die über Todesbescheinigungen identifiziert wurden.

Zur aktuellen Gestaltung der Praxis assistierter Selbsttötung in Deutschland gibt es bislang nur wenige systematische Untersuchungen. Schildmann et al. [6] befragten im März 2021 Mitglieder der Deutschen Gesellschaft für Hämatologie und Onkologie (DGHO) nach ihren klinischen Erfahrungen und Ansichten zur assistierten Selbsttötung. Turiaux et al. [7] führten zwischen Oktober 2023 und Januar 2024 eine webbasierte Umfrage unter den Mitgliedern der Deutschen Gesellschaft für Palliativmedizin (DGP) durch. Für Hausärzt*innen liegt eine S1-Handlungsempfehlung für den Umgang mit dem Wunsch nach Suizidassistenz vor [8].

Seit Februar 2024 arbeiten Vertreter*innen zahlreicher Fachgesellschaften und weiterer Organisationen unter der Federführung der Akademie für Ethik in der Medizin (AEM) – Fachgesellschaft für Ethik im Gesundheitswesen an einer S2k-Leitlinie zum „Umgang mit Anfragen nach Assistenz bei der Selbsttötung“^1^. Grund für diese unter Mitwirkung der Autor*innen dieses Beitrags erfolgte Initiative sind fehlende medizinisch und ethisch begründete Standards für den Umgang mit Anfragen nach Assistenz bei der Selbsttötung. Dringliche Bedarfe für solche Standards bestehen unter anderem bei der Prüfung der Freiverantwortlichkeit sowie der Aufklärung und Beratung bezüglich bestehender alternativer Handlungsoptionen in diesen existenziellen Situationen. Zur empirischen Fundierung der aktuellen Diskussion über eine verantwortbare Praxis der Assistenz bei der Selbsttötung wurde von einem Teil der Autor*innen (AS, GM, JS, SS, TP) eine Berichtsplattform (Bericht- und Lernsystem) „Anfragen und Praxis bezüglich Assistenz bei der Selbsttötung“^2^ initiiert. Seit 01.11.2024 können dort von Fachkräften und Privatpersonen sowohl Fallberichte zu Anfragen nach Assistenz bei der Selbsttötung als auch Informationen zur Praxis der assistierten Selbsttötung eingegeben werden.

Gegenstand dieses Beitrags ist die Darstellung ausgewählter Daten 6 Monate nach Initiierung des Bericht- und Lernsystems (November 2024 bis April 2025). Ziel der Arbeit ist die Darstellung ausgewählter Daten zur Prüfung der Freiverantwortlichkeit und Beratung in der aktuellen Praxis. Ausgehend von dieser Darstellung und unter Berücksichtigung der aktuellen Diskussion über eine verantwortbare Praxis sollen Inhalte möglicher Handlungsempfehlungen für die Prüfung der Freiverantwortlichkeit und Beratung bei Anfragen nach assistierter Selbsttötung erörtert werden.

## Methoden

Das Bericht- und Lernsystem „Anfragen und Praxis bezüglich Assistenz bei der Selbsttötung“ umfasst 26 Fragen zum Umgang mit Anfragen nach assistierter Selbsttötung und angrenzenden Themen, die mittels Einfachauswahl, Mehrfachauswahl sowie teilweise auch mit Freitexten beantwortet werden können. Neben soziodemografischen Daten zu den Berichtenden und den Personen, über die berichtet wird, werden Gründe für Anfragen nach assistierter Selbsttötung, Informationen zur medizinischen und pflegerischen Betreuung sowie Merkmale der Praxis der assistierten Selbsttötung erfragt. Die Entwicklung des Fragebogens erfolgte auf Grundlage empirischer und konzeptioneller Vorarbeiten zum Thema [5, 6, 9, 10].

Informationen über das öffentlich zugängliche Bericht- und Lernsystem und den entsprechenden Fragebogen wurden seit dem 01.11.2024 wiederholt an medizinische Fachgesellschaften sowie weitere Organisationen im Gesundheitswesen versandt. Weiterhin wird über das Bericht- und Lernsystem regelmäßig auf thematisch einschlägigen Veranstaltungen berichtet. Die Abfrage und Eingabe der Daten erfolgen in anonymisierter Form. Eine Unbedenklichkeitsbestätigung der Ethikkommission der Medizinischen Fakultät, Martin-Luther-Universität (MLU) Halle-Wittenberg wurde eingeholt (Reg. No. 2024-033). Die Auswertung der quantitativen Daten erfolgt deskriptiv. Die Freitextantworten wurden entsprechend ihrem Inhalt von 2 Personen kategorisiert (JS, ED). Es wurden nur Berichte von Personen in die Auswertung eingeschlossen, die Angaben zu wenigstens den ersten 4 Fragen im Bericht- und Lernsystem gemacht hatten.

## Ergebnisse

### Berichtende Personen.

Von den 206 Eintragungen im Bericht- und Lernsystem konnten nach Anwendung des Ausschlusskriteriums 133 Fallberichte zu (Anfragen nach) assistierter Selbsttötung ausgewertet werden. Bei einem Teil der Fragen handelt es sich um Filterfragen, weiterhin war die Beantwortung der Fragen freiwillig, sodass nicht alle Fragen von allen Berichtenden beantwortet wurden. Die Berichte stammten am häufigsten von Ärzt*innen (*n* = 70), Pflegefachpersonen (*n* = 18) sowie Ethikberater*innen (*n* = 15; Tab. 1). Dabei konnte die Mehrzahl der Antwortenden sich unter bestimmten Bedingungen vorstellen, eine Assistenz bei der Selbsttötung zu leisten (*n* = 86, 65 %), gut ein Viertel würde dies selbst nicht tun (*n* = 34, 26 %).

**Tab. 1:** Rolle der berichtenden Personen bei der Anfrage nach assistierter Selbsttötung (Mehrfachantworten möglich, Anzahl der antwortenden Personen: *n* = 133)

Antwort	Anzahl
Ärzt*in	70
Pflegefachperson	18
Ethikberater*in	15
Hospizmitarbeiter*in	9
Angehörige*r	7
Andere therapeutische Berufe	4
Seelsorger*in	3
Sozialarbeiter*in	3
Ehrenamtlich Beteiligte*r (Hospizverein, Besuchsdienst etc.)	3
Freund*in/Bekannte*r	3
Psycholog*in/Psychotherapeut*in	2
Jurist*in	0
Sonstiges	14

### Soziodemografische Merkmale der Anfragenden.

Bei 61 Personen, die nach assistierter Selbsttötung anfragten, handelte es sich um Männer, 58 waren Frauen. 71 Personen waren älter als 70 Jahre, 14 Personen waren 50 Jahre oder jünger. 57 Personen waren alleinstehend, 46 waren verheiratet beziehungsweise lebten in einer Partnerschaft.

### Vorhandene Erkrankungen und Gründe für Anfragen.

In 38 Fällen lag eine Krebserkrankung vor, in 36 Fällen eine neurologische Erkrankung und in 16 Fällen eine psychische Erkrankung, wobei für einen Teil der Personen auch mehr als eine Erkrankung berichtet wurde. In 12 Fallberichten wurde angegeben, dass keine Erkrankung vorlag. Die Sorge vor Verlust der Selbstbestimmung (*n* = 33) beziehungsweise der Selbstständigkeit (*n* = 20) wurden am häufigsten als Gründe für die Anfrage nach assistierter Selbsttötung genannt (Tab. 2).

**Tab. 2:** Gründe für die Anfrage nach assistierter Selbsttötung (Anzahl der antwortenden Personen: *n* = 118)

Antwort	Anzahl
Sorge vor Verlust der Selbstbestimmung	33
Sorge vor Verlust der Selbstständigkeit	20
Körperliche Symptome (außer Schmerzen)	13
Schmerzen	9
Psychische Symptome	8
Sorge vor Verlust der Würde	8
Vereinsamung	3
Sorge, eine Last für Familie/Freund*innen zu sein	3
Hohes Alter	2
Schlafstörungen	1
Weiß nicht	0
Sonstiges	8
Keine Antwort	10

### Praxis der assistieren Selbsttötung.

In 22 Fallberichten wurden Angaben zur assistierten Selbsttötung und in weiteren 18 Fallberichten zu einer geplanten assistierten Selbsttötung gemacht. Als Zeitraum zwischen der ersten Anfrage und der assistierten Selbsttötung wurde in 12 Fallberichten weniger als 3 Monate und in 9 Fallberichten mehr als 4 Monate angegeben.

### Prüfung der Freiverantwortlichkeit und Beratung.

In die Prüfung der Freiverantwortlichkeit waren am häufigsten Ärzt*innen und Mitarbeitende von Sterbehilfeorganisationen einbezogen (Tab. 3). In 7 Fallberichten wurde angegeben, dass 2 unabhängige Personen die Freiverantwortlichkeit geprüft hatten. Entsprechend den Angaben in den Freitexten zur Vorgehensweise bei der Prüfung der Freiverantwortlichkeit wurden in 13 Fallberichten „mehrere Gespräche“ als ein Merkmal genannt. In 7 Fallberichten wurde auf die Erstellung eines „Gutachtens oder vergleichbaren Berichts“ verwiesen, in 6 Fällen wurde die „Abklärung von Handlungsalternativen“ und in 5 Fallberichten die „Prüfung der psychischen Gesundheit“ als Merkmale der Prüfung von Freiverantwortlichkeit genannt. In 15 Fallberichten wurden Ärzt*innen genannt, die über verfügbare Handlungsoptionen informiert haben, in 10 Fallberichten wurden Mitglieder von Sterbehilfeorganisationen genannt.

**Tab. 3:** Personen, die die Freiverantwortlichkeit der Anfragenden geprüft haben (Mehrfachantworten möglich, Anzahl der antwortenden Personen: *n* = 39)

Antwort	Anzahl
Ärzt*in	30
Mitarbeiter*in einer Sterbehilfeorganisation	13
Jurist*in	8
Zwei voneinander unabhängige Personen	7
Angehörige*r	5
Pflegefachperson	4
Psychotherapeut*in	1
Eine Person, die nicht in die Information/Beratung einbezogen war	1
Nicht bekannt	0
Andere	7

### Herausforderungen, unterstützende Aspekte, Bedarfe.

Als problematische Aspekte im Kontext von assistierter Selbsttötung wurden Reaktionen von Professionellen (*n* = 9) beziehungsweise aus dem privaten Umfeld (*n* = 7) am häufigsten genannt. In 19 Fallberichten wurden etwaige problematische Aspekte verneint. Raum und Zeit für ausführliche Gespräche (*n* = 64), eine vertiefte Beschäftigung mit dem Thema (*n* = 64) sowie die Gewissheit, dass die anfragende Person sich ihrer Entscheidung sicher ist, wurden als unterstützende Aspekte genannt. In Bezug auf bestehende Bedarfe wurden klare rechtliche Regelungen (*n* = 33) und fachliche Leitlinien (*n* = 20) am häufigsten genannt. 43 Personen gaben an, dass kein weiterer Unterstützungsbedarf bestehe.

## Diskussion

Die vorstehend vorgestellten Daten erlauben keine repräsentativen Aussagen über die Praxis des Umgangs mit (Anfragen nach) assistierter Selbsttötung. Die Berichterstattung erfolgte freiwillig, sodass von Selektionseffekten auszugehen ist. Die Berichte geben aber einen Einblick in die aktuelle Praxis des Umgangs mit Anfragen nach assistierter Selbsttötung und Assistenz bei der Selbsttötung. Ausgehend von den vorliegenden Befunden zur Prüfung der Einsichts- und Urteilsfähigkeit sowie zu Information und Beratung sollen im Folgenden mögliche inhaltliche und prozedurale Anforderungen erörtert werden.

### Prüfung der Einsichts- und Urteilsfähigkeit

In Bezug auf die Prüfung der Einsichts- und Urteilsfähigkeit weisen die erhobenen Informationen zunächst auf Bedarfe hinsichtlich inhaltlicher Anforderungen und Qualifizierung der Prüfenden hin. So wird in 16 von 40 Fällen von einer psychischen Erkrankung berichtet. Während unstrittig ist, dass psychische Erkrankungen einen Einfluss auf die Einsichts- und Urteilsfähigkeit haben können, ist derzeit unklar [11], in welchen Situationen eine Fachperson mit psychiatrischer bzw. psychotherapeutischer Expertise in die Prüfung der Freiverantwortlichkeit einbezogen werden sollte. In den Untersuchungen von Gleich et al. [5] war nur in der Hälfte der Fälle mit psychiatrischen Erkrankungen (Depression, kognitive Einschränkung, Demenz) ein psychiatrisches bzw. psychologisches Fachgutachten dokumentiert. Die Diagnose einer psychischen Erkrankung könnte ein Anlass für die Einbeziehung psychiatrischer bzw. psychologischer Expertise sein. Dabei ist aber zu bedenken, dass auch ohne eine solche Diagnose relevante Beeinträchtigungen der Urteils- und Einsichtsfähigkeit bestehen können. Auf der anderen Seite schließt eine psychische Erkrankung für sich genommen eine freiverantwortliche Entscheidung über die Selbsttötung nicht aus, sie erhöht nur die Wahrscheinlichkeit, dass Betroffene keine informierte selbstbestimmte Entscheidung treffen können [12]. Die Unterstützung durch strukturierte Leitfäden zur Prüfung von Urteils- und Einsichtsfähigkeit [13] sowie die Qualifizierung von Ärzt*innen für die Prüfung der Freiverantwortlichkeit könnten einen Beitrag leisten, inhaltliche Anforderungen, wie sie in Leitlinien festgeschrieben werden können, in der Praxis umzusetzen.

Die erhobenen Informationen aus dem Bericht- und Lernsystem zur Prüfung der Freiverantwortlichkeit machen auch deutlich, dass prozedurale Aspekte der Freiverantwortlichkeitsprüfung in einer Leitlinie und vergleichbare Regelungen bedacht werden sollten. Der Zeitraum zwischen der ersten Anfrage einer sterbewilligen Person und der Assistenz bei der Selbsttötung wird in immerhin 12 von 22 Fallberichten mit weniger als 3 Monaten angegeben. In der Studie von Gleich et al. [14] lag der Zeitraum zwischen erstelltem Gutachten und der Durchführung der assistierten Selbsttötung bei durchschnittlich 23 Tagen (Median 7 Tage, Min. 1 Tag, Max. 106 Tage). Gemessen an den Anforderungen bei der Prüfung der Einsichts- und Urteilsfähigkeit, der Vermittlung entscheidungsrelevanter Informationen sowie der Prüfung der Dauer- und Ernsthaftigkeit erscheint dies auf den ersten Blick als eine eher kurze Zeitspanne. Zudem stellt sich die Frage, welche Bedenkzeit den Betroffenen nach der Prüfung der Freiverantwortlichkeit sowie Beratung und vor der Umsetzung der assistierten Selbsttötung erforderlich ist. Dabei muss bedacht werden, dass sich die Anfragenden in ganz unterschiedlichen Lebenssituationen befinden, wenn sie eine assistierte Selbsttötung für sich erwägen. Es ist zu erwägen, ob bei einer begrenzten Lebenserwartung die Anforderungen an den zeitlichen Abstand und die Häufigkeit der Prüfung der Freiverantwortlichkeit sowie der Ernsthaftigkeit des Sterbewunsches anders definiert werden als in Situationen, in denen Menschen ohne eine lebenslimitierende Erkrankung eine assistierte Selbsttötung anfragen.

Ein weiteres wichtiges prozedurales Merkmal, das bei einer Leitlinie bedacht werden muss, ist die personelle Trennung zwischen Beratung, Prüfung der Freiverantwortlichkeit und der eigentlichen Assistenz bei der Selbsttötung sowie der Leichenschau. In der Untersuchung von Gleich et al. [5] übernahm in der ersten Studienphase in 17 von 37 Fällen ein Arzt alle Aufgaben, von der Arzneistoffverordnung über die Gutachtenerstellung bis hin zu Assistenz bei der Durchführung und Leichenschau. Auch in der Studie von Turiaux et al. [7] lagen bei 4 von 10 assistierten Selbsttötungen Begutachtung und Assistenz in einer Hand. Sowohl aus fachlichen Gründen als auch mit Blick auf mögliche Interessenkonflikte erscheint insbesondere die Prüfung der Freiverantwortlichkeit durch wenigstens 2 voneinander unabhängige Personen unabdingbar. Fachlich verbessert ein solches Vorgehen insbesondere dann die Qualität der Prüfung, wenn die beteiligten Personen unterschiedliche Aspekte, wie etwa die Kenntnis entscheidungsrelevanter Faktoren oder den Ausschluss psychischer Beeinträchtigungen, abdecken. In Bezug auf Interessenkonflikte sind unter anderem finanzielle Interessen zu bedenken, die das professionelle Urteil bei der Prüfung der Freiverantwortlichkeit verzerren können.

### Information und Beratung

Wie bereits erwähnt weisen die Fallberichte darauf hin, dass die Anfragen nach assistierter Selbsttötung im Kontext eines breiten Spektrums an Lebenssituationen gestellt werden. Entsprechend unterschiedlich sind auch die Motivationen für eine assistierte Selbsttötung. Im vorliegenden Berichtssystem wurden vor allem Sorgen vor Verlust der Selbstständigkeit, der Selbstbestimmung bzw. der Würde genannt, zudem Schmerzen und andere körperliche Symptome (Tab. 2). In der Studie von Gleich et al. [5] wurden u. a. chronische Erkrankungen, chronische Schmerzen, Seh- und Hörstörungen, Frailty-Syndrom, Angst vor Pflegebedürftigkeit bzw. eine bestehende Pflegebedürftigkeit genannt. Dies ist für Information und Beratung insofern relevant, als dass in Abhängigkeit von der jeweiligen Situation und Motivationslage der Betroffenen ganz unterschiedliche Handlungsalternativen bedacht beziehungsweise bei der Beratung berücksichtigt werden müssen. Auch hier scheint es in der aktuellen Praxis Verbesserungsbedarf zu geben: In immerhin knapp einem Viertel der Fälle gaben die Befragten in der Studie von Turiaux et al. [7] an, die sterbewillige Person sei nicht (ausreichend) über existierende Hilfemöglichkeiten informiert gewesen.

Eine erste, auch in der öffentlichen Diskussion häufig genannte Konstellation, in der assistierte Selbsttötung angefragt wird, betrifft Menschen, die beispielsweise aufgrund einer Krebserkrankung nicht mehr lange zu leben haben und über den Todeszeitpunkt selbst bestimmen möchten. Eine andere Konstellation umfasst Menschen, die hochaltrig, gebrechlich, häufig alleinstehend und in ihren Fähigkeiten zur Gestaltung des Alltags eingeschränkt sind. Entsprechend den von der DGHS für 2024 berichteten Zahlen war mehr als die Hälfte der Sterbewilligen 80 Jahre oder älter [15]. Menschen mit lebensbegrenzenden Erkrankungen und belastenden Symptomen haben in Deutschland glücklicherweise Anspruch auf eine palliativmedizinische Versorgung. In vielen Fällen verschwinden Wünsche nach einem vorzeitigen Tod unter einer umfassenden Symptomkontrolle in Verbindung mit psychologischer, sozialer und spiritueller Begleitung. Eine qualifizierte Information und Beratung bei Anfragen nach assistierter Selbsttötung müssen in solchen Fällen sicherstellen, dass neben den auf die Erkrankung gerichteten Handlungsoptionen – sofern verfügbar – auch fundiert über die Möglichkeiten der Palliativversorgung bzw. Schmerztherapie und den Zugang hierzu informiert wird. Die verfügbaren Daten weisen darauf hin, dass dies bislang offenbar nicht in allen Fällen gewährleistet ist [16].

Andere Anforderungen ergeben sich bei Information und Beratung von hochaltrigen Menschen ohne spezifische Erkrankung. Medizinische Unterstützungsangebote zur Verbesserung der Gesundheit bzw. Symptomlinderung stehen hier meist nicht im Vordergrund. Einsamkeit, fehlende Zuwendung, Armut oder Pflege, die aus welchen Gründen auch immer als nicht ausreichend beziehungsweise nicht angemessen empfunden wird, können Treiber für den Wunsch nach einem vorzeitigen Tod sein. Es ist vor diesem Hintergrund richtig und wichtig, im Rahmen der Beratung zu prüfen, welche Unterstützungsangebote im psychosozialen Bereich gemacht werden können. Hierbei sollten ggf. auch weitere Berufsgruppen, wie z. B. Sozialarbeiter*innen, hinzugezogen werden.

Eine besondere Herausforderung dürfte zudem darin bestehen, denjenigen Personen eine effektive Unterstützung anzubieten, die sich aufgrund chronischer Erkrankungen oder zunehmender Gebrechlichkeit um den Verlust der Würde, der Selbstbestimmung bzw. der Selbstständigkeit sorgen, die nach den hier berichteten Ergebnissen und der Umfrage von Turiaux et al. [7] einen großen – wenn nicht sogar den größten – Anteil der Anfragen nach assistierter Selbsttötung ausmachen dürften.

Ergänzend zu inhaltlichen Aspekten sind mit Blick auf eine Leitlinie auch Empfehlungen zur Vorgehensweise bei der Beratung zu bedenken. Hier gilt es sicherzustellen, dass die primäre Zielsetzung der Beratung sich nicht an inhaltlichen Positionen der Beratenden für oder gegen die assistierte Selbsttötung orientiert, sondern daran, dass Menschen in die Lage versetzt werden, selbstbestimmt eine Entscheidung zu treffen. Diese Zielsetzung spiegelt sich im Bericht- und Lernsystem dahin gehend wider, dass viele Berichtende die Entscheidungssicherheit aufseiten der Anfragenden als wichtigen unterstützenden Aspekt benennen. Wie bei medizinischen Behandlungsentscheidungen, die dem Ideal einer gemeinsamen Entscheidungsfindung folgen, sollten sich die Gespräche mit den sterbewilligen Personen deshalb nicht auf die Übermittlung entscheidungsrelevanter Informationen beschränken. Vielmehr sollten die Betroffenen auch darin unterstützt werden, die verschiedenen Handlungsoptionen gegeneinander abzuwägen und dann eine Entscheidung zu treffen, die ihren individuellen Präferenzen und ihrer Lebenssituation bestmöglich entspricht [17]. Für die kompetente Gesprächsführung und Begleitung der Betroffenen ist eine entsprechend ausgewiesene Kompetenz der Professionellen im Umgang mit Sterbewünschen unverzichtbar [18].

## Fazit

Die hier vorgestellte vorläufige Auswertung der Daten des Bericht- und Lernsystems geben einen nicht repräsentativen Einblick in die Praxis der Anfragen nach Assistenz bei der Selbsttötung im Zeitraum von November 2024 bis April 2025. Die Angaben der teilnehmenden Personen weisen darauf hin, dass die aktuelle Praxis insbesondere auch hinsichtlich der Prüfung der Einsichts- und Urteilsfähigkeit sowie Information und Beratung unterschiedlich gestaltet und in mancher Hinsicht problematisch ist. Zudem bieten die Informationen Anknüpfungspunkte für die Diskussion über fachliche Anforderungen, die an Akteure gestellt werden müssen, die mit Anfragen nach assistierter Selbsttötung konfrontiert werden. Neben klaren rechtlichen Regelungen wünschten sich die teilnehmenden Personen vor allem auch fachliche Leitlinien sowie psychologische Unterstützung und Supervision. Für einen verantwortlichen Umgang mit Anfragen nach Assistenz bei der Selbsttötung erscheint es deshalb wichtig, entsprechende fachliche Standards auszuarbeiten, die die Qualität der Beratung, sowie der Prüfung und Sicherstellung der Freiverantwortlichkeit gewährleisten. Dies fördert nicht nur die Selbstbestimmung der anfragenden Personen, sondern auch die Rechtssicherheit der assistierenden Personen. Angesichts der unterschiedlichen Lebenssituationen und Motivationen zur assistierten Selbsttötung sind sowohl bei der Prüfung der Einsichts- und Urteilsfähigkeit als auch bei Information und Beratung unterschiedliche Fachkenntnisse erforderlich. Entsprechende inhaltliche und prozedurale Anforderungen werden aktuell in einer S2k-Leitlinie zum Umgang mit Anfragen nach Assistenz bei der Selbsttötung erarbeitet und mit den beteiligten wissenschaftlichen Fachgesellschaften und Organisationen abgestimmt.

## Data Availability

Die während der vorliegenden Studie erzeugten und analysierten Datensätze sind aufgrund potenziell identifizierbarer Informationen nicht öffentlich zugänglich. Weitere Informationen können bei den Autor*innen angefordert werden.

## Footnotes

1 Registrierung: https://register.awmf.org/de/leitlinien/detail/096-001. Zugegriffen: 10.10.2025.

2 https://www.forschungsnetzwerk-suizidassistenz.de/register/. Zugegriffen: 10.10.2025.
